# Multi-regional transcriptomic profiling reveals divergent molecular mechanisms in ALS-related neurodegeneration

**DOI:** 10.1371/journal.pgen.1012225

**Published:** 2026-07-08

**Authors:** Yu-Wen Hsu, Yu-Ning Lu, Mingming Liu, Jiou Wang

**Affiliations:** 1 Department of Biochemistry and Molecular Biology, Bloomberg School of Public Health, Johns Hopkins University, Baltimore, Maryland, United States of America; 2 Department of Neuroscience, School of Medicine, Johns Hopkins University, Baltimore, Maryland, United States of America; HudsonAlpha Institute for Biotechnology, UNITED STATES OF AMERICA

## Abstract

Neurodegenerative disorders including amyotrophic lateral sclerosis (ALS) remain largely unsolved, with complex etiology yet to be fully elucidated. The most common genetic cause of ALS in both familial and sporadic cases is the expansion of a hexanucleotide repeat in the *C9orf72* gene. To systematically dissect the molecular landscape of ALS, we performed integrative transcriptomic analyses across multiple central nervous system regions from ALS patients carrying pathological *C9orf72* repeat expansions (ALS-C9) and those without the mutation (ALS-non-C9). In parallel, we performed transcriptome-wide cell-type deconvolution to assess the cellular composition of neuronal and non-neuronal populations. We identified a set of dysregulated molecular pathways that were consistently altered in both ALS-C9 and ALS-non-C9 patients, suggesting shared pathogenic mechanisms. Distinct gene-specific alterations also pointed to divergent subtype-dependent molecular trajectories. Gene-specific alterations were also associated with short clinical duration in ALS-non-C9, highlighting a sex-dependent immunological contribution to disease outcome. Our cross-regional integrative transcriptomic analyses reveal both convergent and divergent molecular and cellular features between ALS-C9 and ALS-non-C9 subgroups, underscoring the clinical heterogeneity of ALS and providing a framework for subtype- and sex-specific therapeutic stratifications.

## Author summary

Amyotrophic lateral sclerosis (ALS) is a fatal neurodegenerative disease characterized by the progressive loss of motor neurons. While mutations in the *C9orf72* gene represent the most common genetic cause of ALS, the molecular differences between *C9orf72* mutation carriers and non-carriers remain incompletely understood, particularly across different brain and spinal cord regions. In this study, we analyzed postmortem brain and spinal cord tissue from ALS patients with and without *C9orf72* mutations alongside neurologically normal controls, profiling gene expression across ten distinct regions of the central nervous system. We identified both shared and genotype-specific transcriptional signatures, showing that, despite these common features, ALS-C9 and ALS-non-C9 engage distinct molecular programs not only in disease pathogenesis but also in relation to the rate of clinical decline. Pathways broadly activated in the ALS disease state showed opposing directionality between short- and long-duration cases in the two genetic subgroups, with immune and metabolic programs being further amplified in short-duration ALS-C9 cases but attenuated in their ALS-non-C9 counterparts. These findings underscore the importance of considering genetic background when investigating ALS disease mechanisms and identifying potential therapeutic targets.

## Introduction

Neurodegenerative diseases are unified by the gradual failure and death of neurons, leading to irreversible impairment of the nervous system functions. Amyotrophic lateral sclerosis (ALS) is a devastating neurodegenerative disorder, marked by the degeneration of upper and lower motor neurons in the brain and spinal cord, which drives decline in motor control, muscle weakness, paralysis, and ultimately respiratory failure. Many ALS cases exhibit an aggressive course, relatively selective neuronal vulnerability, and overlap with frontotemporal dementia (FTD), thus providing a powerful model for uncovering the molecular principles that govern neuronal survival and death. Studying ALS therefore not only informs therapeutic strategies for this fatal disease but also yields insights with broad relevance across the neurodegenerative spectrum.

The most common genetic cause of ALS is the GGGGCC hexanucleotide repeat expansion (HRE) in the *C9orf72* gene. Several pathogenic mechanisms have been implicated in C9orf72-linked ALS/FTD, including loss of functions of C9orf72, RNA toxicity from the repeat-containing RNAs, proteotoxicity from poly-dipeptides produced by the repeat, and DNA-initiated mechanisms [1,2]. *C9orf72* encodes a multi-functional protein involved in endosomal trafficking [3], autophagy [4], immune regulation [5], lipid metabolism [6], and mitochondrial functions [7]. Pathogenic repeat expansions have been shown to disrupt a multitude of cellular functions, including nucleocytoplasmic transport [8], neuroinflammation [5], and global epigenetic regulations [2]. The repeat expansion mutations account for approximately 40% of familial ALS (fALS) and 7% of sporadic ALS (sALS) cases [9]. However, the molecular mechanisms linking C9orf72 to disease heterogeneity and disease duration remain unsolved. The *C9orf72* HRE is also the most common genetic cause for FTD, representing 25% of FTD cases [10]. Although FTD, the second most common dementia in people younger than 65, manifests distinctly from ALS, these two conditions have been recognized as a continuous neurodegenerative spectrum [11].

At the cellular level, ALS is marked by neuronal degeneration and non-neuronal contributions. Degeneration of motor neurons and activation of microglia have been shown to be critical drivers of disease onset and progression rate in ALS models [12,13]. Additionally, reactive astrocytes [14,15] and vessel-associated cells [16,17] contribute to neuroinflammation cascades and blood-brain barrier disruption, further amplify neuronal damage. How molecular mechanisms and cellular compositions contribute to ALS onset and disease duration remains to be elucidated. The extent to which regional transcriptional alterations and cell-type imbalances shape disease heterogeneity and clinical duration requires spatial investigation across the central nervous system.

Given that the *C9orf72* repeat expansion represents the most prevalent genetic alteration in ALS, we conducted transcriptomic profiling and cell-type deconvolutions across multiple regions of the central nervous system in ALS cases stratified by *C9orf72* expansion status. Our analysis revealed a dual picture with shared signatures, such as disruptions in DNA repair, proteostasis, and mitochondrial function, suggesting convergent pathways in ALS pathogenesis, alongside subgroup-specific trajectories. Pathways broadly activated in the ALS disease state, spanning immune, inflammatory, and core metabolic programs, demonstrated a consistent and opposing directionality between short-duration ALS-C9 and ALS-non-C9 patients. Moreover, microglial expansion in the cervical spinal cord correlating with short clinical duration, particularly pronounced in females, implicating sex-dependent mechanisms in neuroinflammatory processes underlying ALS duration. These findings highlight the cellular and molecular heterogeneity of ALS and underscore the importance of incorporating sex as critical biological variable in future mechanistic and therapeutic studies.

## Results

### Sex-dependent survival trend in non-C9orf72 ALS

To comprehensively investigate the molecular landscape of ALS across genetic subtypes, we analyzed transcriptomic data from multiple central nervous system (CNS) regions in 316 subjects, comprising 77 controls and 239 ALS patients. Among the ALS cohorts, 43 patients carried the *C9orf72* pathological repeat expansion (ALS-C9), while the remaining 196 patients did not harbor this mutation (ALS-non-C9). Tissue samples data were obtained from cerebellum, cervical, thoracic, and lumbar spinal cord, lateral and medial motor cortex, frontal cortex, hippocampus, occipital cortex, and temporal cortex.

**Fig 1:**
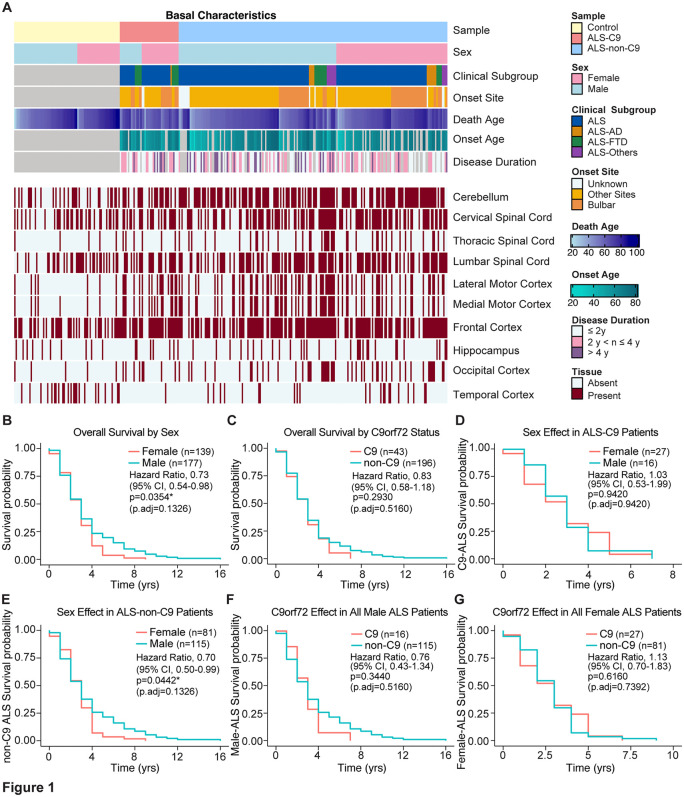
Clinical characteristics and survival profiles of study cohorts. **(A)** A comprehensive heatmap overview of clinical and genetic information across 316 subjects, including 77 controls and 239 ALS patients. The subjects are categorized by sample, sex, clinical subgroup, onset site, death age, onset age, disease duration, and tissue availability across various brain and spinal cord regions. Data regarding onset age and disease duration are not available for 50 ALS patients in the cohort. **(B-G)** Kaplan-Meier curve plot for ALS survival stratified by (B) sex, (C) *C9orf72* genotypes, (D) by sex in ALS-C9 patients, (E) by sex in ALS-non-C9 patients, (F) by *C9orf72* genotypes in male ALS patients, (G) by *C9orf72* genotypes in female ALS patients. Statistical significance is denoted by asterisks: * p < 0.05. Adjusted p-values (p.adj) were calculated using the Benjamini-Hochberg (BH) method to control the false discovery rate (FDR) across multiple comparisons.

We first evaluated phenotypic characteristics between ALS-C9 and ALS-non-C9 patients. The ALS-C9 group had a lower proportion of males (37.2%) relative to ALS-non-C9 patients (58.7%) and controls (59.7%). However, no significant differences were observed between ALS-C9 and ALS-non-C9 groups in clinical parameters, including bulbar onset frequency (37.2% vs. 29.6%, *p* = 0.626), age at symptom onset (59.9 ± 8.7 vs. 60.7 ± 11.4 years old, *p* = 0.3962), age at death (63.4 ± 8.4 vs. 64.7 ± 10.5 years old, *p* = 0.2811), or disease duration (also referred to as survival period, calculated at the age of death minus age at disease onset, 2.8 ± 1.7 vs. 3.3 ± 2.6 years, *p* = 0.6497) (Fig 1A and S1 Table).

Given the sex imbalance, we next examined the independent and interactive effects of sex and *C9orf72* mutations on ALS survival period. Male patients exhibited a modestly extended survival period compared to females (Hazard ratio [HR] = 0.73, 95% CI = 0.54-0.98, *p* = 0.0354, *p.adj* = 0.1326) (Fig 1B). However, no significant survival difference was detected between ALS-C9 and ALS-non-C9 groups (HR = 0.83, 95% CI = 0.58-1.18, *p* = 0.2930, *p.adj* = 0.5160) (Fig 1C).

Stratified analyses revealed no significant sex-based survival differences within the ALS-C9 subgroup (HR = 1.03, 95% CI = 0.53-1.99, *p* = 0.9420, *p.adj* = 0.9420) (Fig 1D). In contrast, male ALS-non-C9 patients demonstrated significantly longer survival period than female counterparts (HR = 0.70, 95% C.I. = 0.50-0.99, *p* = 0.0442, *p.adj* = 0.1326) (Fig 1E). We further performed interaction analyses by stratifying mutation status within each sex group. Neither male nor female subgroups showed significant differences between ALS-C9 and ALS-non-C9 (Fig 1F and 1G).

### Common and divergent transcriptomic changes in C9 and non-C9 ALS

We next conducted transcriptomic analysis across multiple CNS regions, including the cerebellum, cervical, thoracic, and lumbar spinal cord, lateral and medial motor cortex, frontal cortex, hippocampus, occipital cortex, and temporal cortex, to compare gene expression profiles among ALS-C9 patients, ALS-non-C9 patients, and control subjects. Across all tissues, ALS-non-C9 patients showed a higher number of differentially expressed genes (DEGs) compared to ALS-C9 patients. The most prominent transcriptomic alterations were observed in the cervical spinal cord, frontal cortex, and lumbar spinal cord, with over 8,800 DEGs in ALS-non-C9 patients and over 6,700 in ALS-C9 patients (S1 Fig).

**Fig 2:**
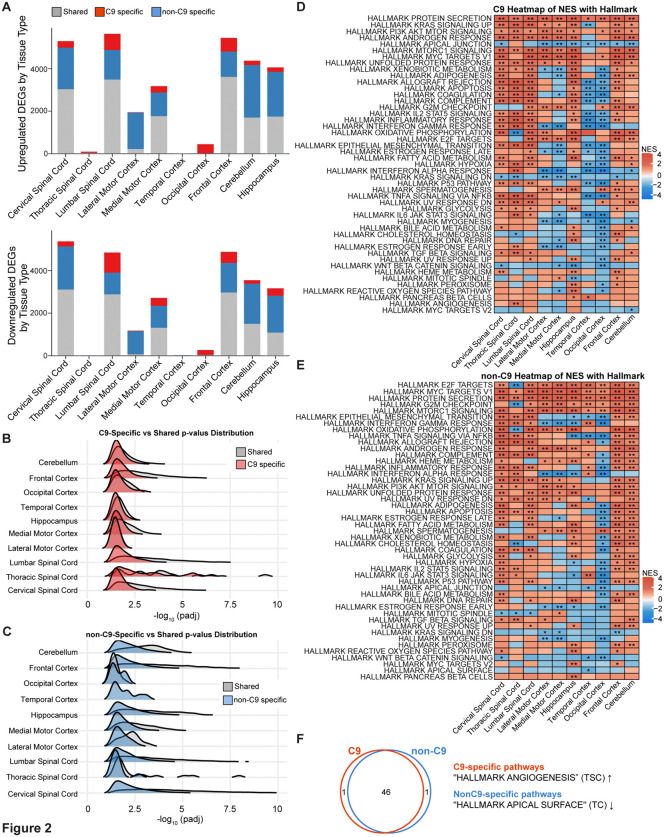
Shared differential gene expression and pathway activation in ALS-C9 and ALS-non-C9 reveal core disease programs. **(A)** Bar plots represent the distribution of significantly upregulated differentially expressed genes (DEGs) (top) and downregulated DEGs (bottom) across tissues. Each bar presents the total amount of significant DEGs in each tissue, classified into three categories: shared DEGs (grey), C9-specific DEGs (red), and non-C9-specific DEGs (blue). **(B)** Ridge plots represent the density distribution of -log10 (p.adj) for DEGs across tissues. Values are capped at 10 on the -log10 (p.adj) scale for visualization purposes, genes exceeding this threshold are displayed at the maximum value. Each lane represents a tissue, with C9-specific DEGs shown in red and C9-shared DEGs shown in grey. **(C)** Ridge plots represent the density distribution of -log10 (p.adj) for DEGs across tissues. Values are capped at 10 on the -log10 (p.adj) scale for visualization purposes, genes exceeding this threshold are displayed at the maximum value. Each lane represents a tissue, with non-C9-specific DEGs shown in blue and non-C9-shared DEGs shown in grey. **(D-E)** Heatmaps depicting the normalized enrichment scores (NES) for hallmark pathways of ALS disease in (D) C9 and (E) non-C9 groups across CNS tissues. Each row represents a pathway, and each column correspond a tissue. NES values indicate pathway directionality: positive NES reflects upregulation in ALS patients relative to controls, while negative NES indicates downregulation. Statistical significance is denoted by asterisks: * p.adj < 0.05, ** p.adj < 0.01, *** p.adj < 0.001. **(F)** Venn diagram summarizes the overlap of significantly enriched pathways between ALS-C9 and ALS-non-C9 groups across all nine tissues including cervical spinal cord (CSC), thoracic spinal cord (TSC), lumbar spinal cord (LSC), lateral motor cortex (LMC), medial motor cortex (MMC), temporal cortex (TC), occipital cortex (OC), frontal cortex (FC), cerebellum (Cere). Upward arrows (↑) indicate upregulation of the corresponding Hallmark pathway, while downward arrows (↓) denote downregulation.

To characterize the tissue-specific landscape of transcriptome changes, we quantified the proportion of DEGs that were specific to ALS-C9, specific to ALS-non-C9, or shared between two groups. In major brain and spinal regions including the frontal cortex (ALS-C9: 15% vs. ALS-non-C9: 28%), cerebellum (ALS-C9: 11% vs. ALS-non-C9: 58%), cervical spinal cord (ALS-C9: 8% vs. ALS-non-C9: 39%), lumbar spinal cord (ALS-C9: 21% vs. ALS-non-C9: 28%), medial motor cortex (ALS-C9: 18% vs. ALS-non-C9: 41%), lateral motor cortex (ALS-C9: 14% vs. ALS-non-C9: 92%), and hippocampus (ALS-C9: 17% vs. ALS-non-C9: 58%), ALS-C9 specific DEGs represented only a small fraction (8–21%), indicating limited transcriptomic specificity in the ALS-C9 group. In contrast, ALS-non-C9 specific DEGs acounted for 28–92% of total DEGs in these regions, reflecting more distinct and widespread gene expression changes. A few regions, such as the occipital cortex and thoracic spinal cord, displayed relatively higher proportions of specific DEGs in ALS-C9 groups, suggesting region-dependent transcriptional alterations. The temporal cortex yielded few significant DEGs across both ALS subgroups. This limited transcriptional response may be attributable to the smaller sample size, which potentially reduced the statistical power to detect subtle molecular change (Fig 2A, S2 Table).

Given the substantially lower proportion of males in the ALS-C9 group relative to ALS-non-C9 and controls, we conducted two complementary analyses to evaluate whether this sex imbalance could drive the observed pathogenesis-associated transcriptomic differences. First, sex-by-diagnosis interaction terms were tested across all tissues in both groups. The proportion of genes with a statistically significant interaction term remained below 0.6% in virtually all tissue-group combinations, with hippocampus representing a minor exception in both ALS-C9 (0.54%) and ALS-non-C9 (0.59%), collectively suggesting that sex-by-diagnosis interactions exert minimal transcriptome-wide influence (S3 Table). Second, we employed the sensitivity analysis to quantify how strongly sex-related confounding would need to operate to invalidate the top 10 ALS-associated DEGs. For individual DEGs, all top 10 genes demonstrated robustness to sex confounding across most tissues in both groups, with temporal cortex in ALS-C9 (20% robust) and thoracic spinal cord in ALS-C9 (70% robust) as notable exceptions (S4 Table). On balance, these findings support the conclusion that the ALS-associated transcriptomic signatures reported here are not substantially driven by sex imbalance, though findings from temporal cortex and thoracic spinal cord in ALS-C9 tissues should be interpreted with appropriate caution.

Additionally, the consistently low proportion of ALS-C9 specific DEGs across most tissues suggest that the majority of transcriptional changes in ALS-C9 are shared with ALS-non-C9. These shared DEGs likely reflect core pathogenic pathways that are common to ALS pathophysiology regardless of genetic background. To further explore their relevance, we compared the statistical significance of subtype-specific versus shared DEGs across multiple CNS regions. The shared DEGs demonstrated stronger statistical significance in both ALS-C9 and ALS-non-C9 patients compared to the subtype-specific DEGs, suggesting their potential roles in driving fundamental molecular mechanisms in ALS (Fig 2B and 2C). To evaluate whether the observed transcriptomic differences between ALS-C9 and ALS-non-C9 could be attributed to sample size discrepancy, we performed a bootstrapping procedure (N = 100 iterations) in which equivalent numbers of samples were drawn from each group to match the smaller ALS-C9 cohort for case-control comparisons. Across most regions, the observed DEG counts fell within or near the bootstrapped distributions, indicating that the magnitude of differential expression signal is broadly consistent with what would be expected at equivalent sample sizes (S2 Fig).

We then performed gene set enrichment analysis (GSEA) separately for the ALS-C9 (Fig 2D) and ALS-non-C9 groups (Fig 2E). Upregulated pathways in both ALS subtypes included protein secretion, KRAS signaling (upregulated), PI3K-AKT-mTOR signaling, E2F targets, G2M checkpoint, unfolded protein response, while downregulated pathways involved KRAS signaling (downregulated). These shared transcriptional signatures suggest a common disruption of proteostasis, cell cycle regulation and proliferative signaling across ALS subtypes, irrespective of genetic etiology. Both groups exhibited largely overlapping enrichment profiles across CNS regions (Fig 2F).

### Contribution of C9 and non-C9 specific genes to distinct pathogenic mechanisms in ALS

**Fig 3:**
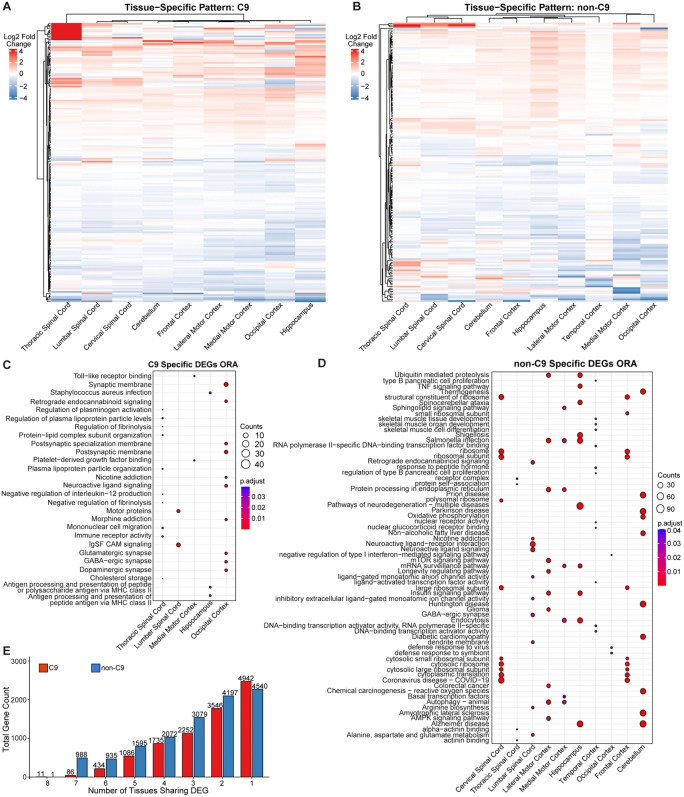
Subtype-specific transcriptomic alterations reveal systemic dysregulation in ALS-non-C9 and tissue-specific heterogeneity in ALS-C9. **(A)** Hierarchical clustering heatmap illustrating the top 20 ALS disease-related differentially expressed genes (DEGs) from each tissue based on the ALS-C9-specific gene sets. This set comprises 178 genes. Each row represents a gene and each column corresponds to a tissue. Log2 fold change values reflect gene expression level, where red indicates upregulation and blue indicates downregulation relative to controls. Temporal cortex is not shown for the ALS-C9 group as no statistically significant differential expression was detected in this region. **(B)** Hierarchical clustering heatmap for the top 20 ALS disease-related DEGs from each tissue based on the ALS-non-C9-specific gene set, which includes 173 genes. The same color scheme is used, with red representing upregulation and blue representing downregulation. **(C)** Dot plot showing the ALS disease-related pathways identified via over-representation analysis (ORA) using GO and KEGG databases, based on the ALS-C9-specific gene sets in each tissue. tissue. Dot size represents the gene count within each enriched pathway, and color indicates the adjusted p-value of enrichment. Only tissues with at least one statistically significant enriched pathway (adjusted p-value < 0.05) are displayed. **(D)** Dot plot showing the ALS disease-related pathways identified via ORA from the ALS-non-C9-specifc gene set in each tissue. Dot size reflects the gene count in each pathway, and color corresponds to the adjusted p-value. **(E)** Bar chart illustrating the distribution of across tissue number of DEGs in ALS-C9 group (red) and ALS-non-C9 group (blue).

To further investigate the divergent molecular mechanisms underlying ALS pathophysiology in ALS-C9 and ALS-non-C9 patients, we selected the top 20 most significant group-specific DEGs per tissue. This yielded 178 DEGs for the ALS-C9 group (S5 Table) and 173 DEGs for the ALS-non-C9 group (S6 Table). Several ALS-C9-specific DEGs, including *APOE* [18], *IGF1* [19], *MAO-B* [20], have been previously been observed to be linked to ALS patients. Similarly, ALS-non-C9-specific DEGs identified in this study, including *ISG15* [21] and *NGFR* [22], have established roles in ALS pathogenesis. Hierarchical clustering of the tissues highlighted distinct differences in anatomical grouping between the two cohorts. The ALS-C9 cohort exhibited highly specialized and fragmented regional expression signature. The unique anatomical compartmentalization disrupted standard tissue clustering, leaving the spinal cord axis split across the dendrogram (Fig 3A). In contrast, tissues in the ALS-non-C9 group demonstrated clear, natural clustering by anatomical region, with all spinal cord tissues grouping tightly together as a distinct branch completely apart from the cortical regions (Fig 3B). Despite these differences in tissue-level clustering, a subset of genes showed consistent directionality across multiple tissues, particularly within the spinal cord axis, while the majority of prioritized DEGs remain tissue-restricted.

Next, we performed over-representation analysis (ORA) on the all subgroup-specific DEGs. The enriched pathways were largely distinct between ALS-C9 and ALS-non-C9 groups. ALS-C9-specific DEGs were predominantly enriched in pathways related to lipid homeostasis, specifically the regulation of plasma lipoprotein particle levels and cholesterol storage. These genes were also enriched in pathways related to immune modulation and collagen metabolism, including Toll-like receptor binding and antigen presentation via MHC class II (Fig 3C). Conversely, the ALS-non-C9-subgroup was characterized by enrichment in pathways associated with protein synthesis. This signature was driven by DEGs involved in cytoplasmic translation, the structural assembly of ribosome and ribosomal subunits. From a disease-association perspective, ALS-non-C9 specific signature showed significant overlap with pathway term associated with other neurodegenerative disorders, most notably Alzheimer’s, Parkinson’s, and Huntington’s diseases (Fig 3D). These results support the existence of both shared and distinct pathogenic mechanisms in ALS-C9 and ALS-non-C9 patients.

To evaluate transcriptomic consistency across CNS regions, we quantified the number of DEGs shared across multiple tissues within each group. ALS-non-C9 patients exhibited markedly higher cross-tissue overlap than ALS-C9 patients. Specifically, 989 DEGs were shared across more than seven CNS regions in the ALS-non-C9 group, whereas only 97 DEGs in the ALS-C9 group. Notably, while the ALS-C9 group exhibited a marginally higher number of DEGs shared across eight tissues, the number of shared DEGs was consistently greater in the ALS-non-C9 group at all other levels of overlap, ranging from two to seven tissues (Fig 3E). Supporting this observation, ALS-C9 patients showed lower counts of overlapping DEGs across CNS tissues. In the ALS-C9 cohort, the most robust transcriptomic signature was shared between the cervical and lumbar spinal cord, representing the highest intersection with 647 upregulated and 703 downregulated genes. The second-highest peaks revealed a broader but more selective distribution. For upregulated genes, a cluster of 266 DEGs was primarily distributed across the cervical spinal cord, lumbar spinal cord, hippocampus, and frontal cortex. Similarly, the second-highest peaks for downregulated genes consisted of 355 DEGs shared across the cervical spinal cord, lumbar spinal cord, and frontal cortex, included the cerebellum instead of the hippocampus (S3A Fig). The ALS-non-C9 patients demonstrated a more extensive degree of sharing across a wider range of tissues. Similar to the ALS-C9 group, the primary peak was found in the cervical and lumbar spinal cord, though with subsequentially higher gene counts of 821 upregulated and 1,081 downregulated genes. The secondary peaks in this cohort showed a significantly more widespread anatomical distribution compared to the ALS-C9 group. Specifically, compared to the second-highest peak observed across four regions in ALS-C9, 659 upregulated and 316 downregulated genes were shard across seven-region network in ALS-non-C9, including the cervical spinal cord, lumbar spinal cord, lateral motor cortex, medial motor cortex, hippocampus, frontal cortex, and cerebellum (S3B Fig). These results suggest a more consistent and system-wide transcriptomic alteration in ALS-non-C9, whereas ALS-C9 demonstrates more tissue-specific and heterogeneous gene expression changes.

### Divergent pathway directionality between ALS-C9 and ALS-non-C9 across clinical duration groups

Lifespan variability is a notable characteristic among ALS patients, with clinical durations in our cohort ranging from less than one year to up to 16 years. To investigate transcriptomic differences associated with disease duration, we categorized ALS patients into distinct duration groups based on the quartiles of the disease duration distribution across the cohort. Patients with a disease duration falling at or below the first quartile (≤2 years) were defined as the short clinical duration group, while those at or above the third quartile (≥4 years) were defined as the long clinical duration group, effectively contrasting the extreme survival phenotypes while excluding intermediated durations. Differential expression analysis revealed a relatively small number of duration-associated DEGs across most tissues in both ALS-C9 and ALS-non-C9 group (S7 Table). A notable exception was observed in the ALS-C9 cerebellum, which exhibited a substantially higher counts of DEGs compared to other CNS regions. Diagnostic examination of the cerebellum dispersion estimates showed that final estimates were tightly clustered around the fitted trend line with a smooth fit, confirming that the high DEG count did not result from technical problems with the model fitting itself (S4 Fig). Following this quality validation, we conducted GSEA to characterize functional pathway-level patterns associated with clinical duration.

**Fig 4:**
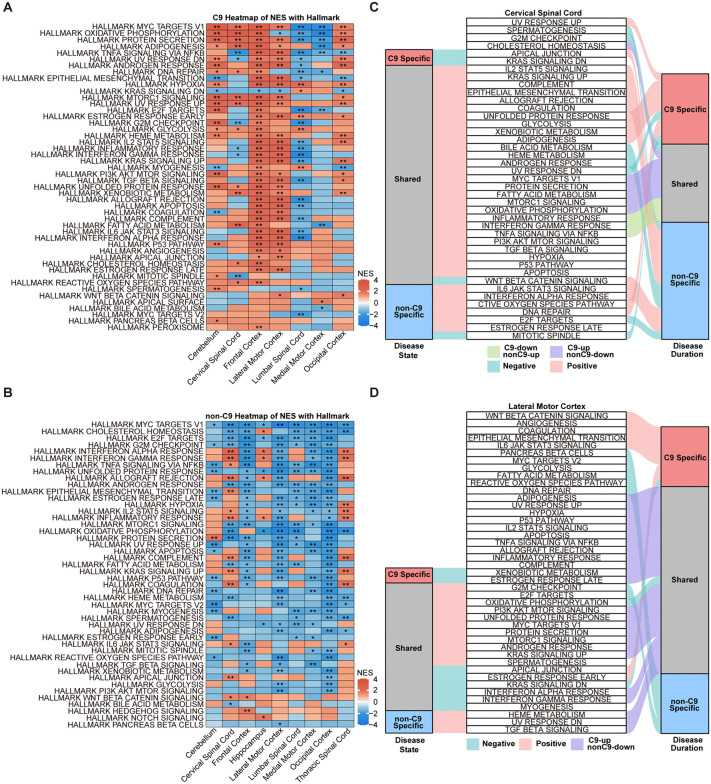
Differential disease druration signatures and pathway transition landscapes in ALS-C9 and ALS-non-C9. **(A)** Heatmaps depicting the normalized enrichment scores (NES) for hallmark pathways of ALS clinical duration in ALS-C9 group across different tissues. No significant pathways were identified in the thoracic spinal cord, hippocampus and temporal cortex in ALS-C9 group. Therefore, these tissues are not displayed in the figure. NES values indicate pathway directionality: positive NES reflects upregulation in ALS short-duration patients relative to long-duration patients, while negative NES indicates downregulation. Statistical significance is denoted by asterisks: * p.adj < 0.05, ** p.adj < 0.01. **(B)** Heatmaps depicting the normalized enrichment scores (NES) for hallmark pathways of ALS clinical duration in ALS-non-C9 group across different tissues. No significant pathways were identified in the temporal cortex in ALS-non-C9 group. Therefore, these tissues are not displayed in the figure. NES values indicate pathway directionality: positive NES reflects upregulation in ALS short-duration patients relative to long-duration patients, while negative NES indicates downregulation. Statistical significance is denoted by asterisks: * p.adj < 0.05, ** p.adj < 0.01. **(C-D)** Sankey diagrams illustrate transitions in pathways enrichment specificity across the axes of ALS disease state specificity, pathway identity, and disease duration specificity across (C) cervical spinal cord, and (D) lateral motor cortex. Pathways are categorized into three specificity groups based on their enrichment profiles: C9-specific, non-C9-specific, or shared (significant in both groups). The direction of NES is visualized through color-coded flows: red for up regulation (positive NES), blue for downregulation (negative NES), light green for ALS-C9 down regulation but ALS-non-C9 upregulation, purple for ALS-C9 upregulation but ALS-non-C9 downregulation.

Pathway enrichment analysis revealed divergent duration-related trend between ALS-C9 and ALS-non-C9 patients across CNS regions. In ALS-C9 patients, short clinical duration was associated with broad upregulation of proliferative, metabolic, and immune-related pathways across most tissues relative to long clinical duration, including MYC targets, oxidative phosphorylation, mTORC1 signaling, interferon responses, inflammatory response, complement activation, TNFα signaling, apoptosis, and DNA repair. Additional selective upregulation of hypoxia, glycolysis, IL6-JAK-STAT3, and angiogenesis pathways was observed, collectively pointing to a metabolic and hypoxic stress signature characteristic of short-duration ALS-C9 cases (Fig 4A). Strikingly, the directionality of these pathway enrichments was reversed in ALS-non-C9 patients when evaluating short-duration individuals against long-duration groups. In this ALS-non-C9 cohort, short clinical duration was instead associated with consistent and widespread downregulation of the same pathway set across nearly all tissues examined, encompassing both spinal cord and cortical regions (Fig 4B). To distinguish transcriptomic alterations associated with disease state (ALS patients vs. controls) from those linked to disease duration (short-duration vs. long-duration), we applied Sankey diagram analysis for a detailed examination of subtype-specific pathway transitions.

In the cervical spinal cord, among pathways shared in the ALS disease state, a subset including inflammatory response, interferon gamma response and TNFα signaling via NFκB transitioned to ALS-C9-specific downregulation in patients with short clinical duration relative to those with long clinical duration. This pattern suggests that these immune and inflammatory programs, while broadly activated in ALS pathogenesis, are relatively suppressed in short-duration ALS-C9 cohort when contrasted with their long-duration counterparts. Conversely, pathways such as MYC target v1, protein secretion, fatty acid metabolism, mTORC1 signaling, and oxidative phosphorylation followed the opposite trajectory. While remaining shared and elevated in the disease state overall, they became specifically repressed in short-duration ALS-non-C9 patients when compared directly to long-duration individuals. This indicates that core metabolic and biosynthetic programs are preferentially attenuated in rapid-progressing short-duration ALS-non-C9 cases relative to the long duration baseline (Fig 4C).

In the lateral motor cortex, WNT-β-catenin signaling, angiogenesis, coagulation, epithelial-mesenchymal transition, and IL6-JAK-STAT3 signaling were selectively upregulated in the ALS-C9 short clinical duration group compared to the ALS-C9 long clinical duration group. A broader set of pathways upregulated in both ALS subgroups at the disease state level exhibited divergent directionality in the clinical duration comparison, remaining upregulated in ALS-C9 short duration while becoming downregulated in ALS-non-C9 short duration, with both groups evaluated against their respective long-duration baselines. This divergent pattern including proliferative and cell cycle programs (MYC targets V1, mTORC1 signaling, PI3K-AKT-mTOR signaling), protein homeostasis pathways (protein secretion, unfolded protein response), and metabolic and hormonal regulators (androgen response, KRAS signaling) (Fig 4D).

This divergent directionality pattern was consistently reproduced across the lateral motor cortex and frontal cortex, where immune and inflammatory programs encompassing interferon alpha response, interferon gamma response, and inflammatory response, stress and damage response pathways including p53 pathway, apoptosis, UV response, and TNFα signaling via NFκB, and metabolic regulators such as heme metabolism, hypoxia, and estrogen response late, were all upregulated in ALS-C9 short duration but downregulated in ALS-non-C9 short duration, always using long-duration patients as the reference group. The reproducibility of this pattern across both lateral motor cortex and frontal cortex regions suggests that the opposing transcriptional responses to clinical duration between the two genotypes reflect a broad and consistent molecular divergence rather than a region-specific phenomenon.

In the frontal cortex, this cross-directional pattern was further extended to encompass additional cell cycle and metabolic programs, including G2M checkpoint, E2F targets, fatty acid metabolism, and cholesterol homeostasis, alongside the shared pathways described above, with consistent upregulation in ALS-C9 short duration and downregulation in ALS-non-C9 short duration relative to long-duration individuals (S5A Fig).

Beyond these three regions, a similar cross-directional pattern was observed in additional regions. In the lumbar spinal cord, myogenesis, estrogen response early, and hypoxia followed the same divergent directionality, being upregulated in both ALS subgroups at the disease state level yet selectively upregulated in ALS-C9 short duration and downregulated in ALS-non-C9 short duration when compared to long-duration cases. In the cerebellum, this pattern extended to cell cycle and stress response programs, including G2M checkpoint, MYC targets V1, p53 pathway, and UV response, while the occipital cortex exhibited divergent directionality across a broader set of programs encompassing proliferative signaling (MYC targets V1), secretory function (protein secretion), metabolic regulation (adipogenesis), and cellular stress responses (hypoxia, IL2-STAT5 signaling), with all changes reflecting the contrast of short- versus long-duration cohorts. Of note, no significant pathway enrichment associated with clinical duration was identified in the temporal cortex in either group, a finding attributable to the limited sample size available for this region, which precluded reliable detection of duration-associated transcriptional signals (S5B-S5H Fig, S8 Table).

Together, these results underscore fundamental transcriptomic differences between ALS-C9 and ALS-non-C9. Pathways broadly activated in the ALS disease state, including immune and inflammatory programs such as interferon responses, inflammatory response, and TNFα signaling via NFκB, as well as core metabolic and biosynthetic programs such as MYC targets V1, mTORC1 signaling, oxidative phosphorylation, and protein secretion, showed divergent behavior in the clinical duration comparison. These pathways were selectively upregulated in short-duration ALS-C9 cases while being downregulated in short-duration ALS-non-C9 cases, demonstrating that when short-duration individuals are compared directly to long-duration patient, the relative transcriptional differences move in opposite directions between the two genotypes. This divergence indicates that while the two genetic subgroups share a broadly similar pathogenic transcriptional landscape, the molecular signature associated with a rapid disease course (short duration) relative to slower disease course (long-duration) is fundamentally inverted in ALS-C9 and ALS-non-C9, pointing to distinct biological mechanisms underlying patient survival times in these groups.

### APOC2 is positively correlated with microglia and upregulated in females, potentially contributing to shorter disease duration in Non-C9 ALS-patients

To investigate cellular composition and its impact on disease onset and disease duration in ALS-C9 and ALS-non-C9 patients, we applied CIBERSORTx-based cell deconvolution using reference single-cell expression data [23]. This approach categorized cellular signatures into three groups: neuronal cells (neuronal progenitor, excitatory neuron, inhibitory neuron), glia cells (glial progenitor, astrocyte, microglia), and vascular cells (endothelial cells, mural cells, choroid plexus cells).

**Fig 5:**
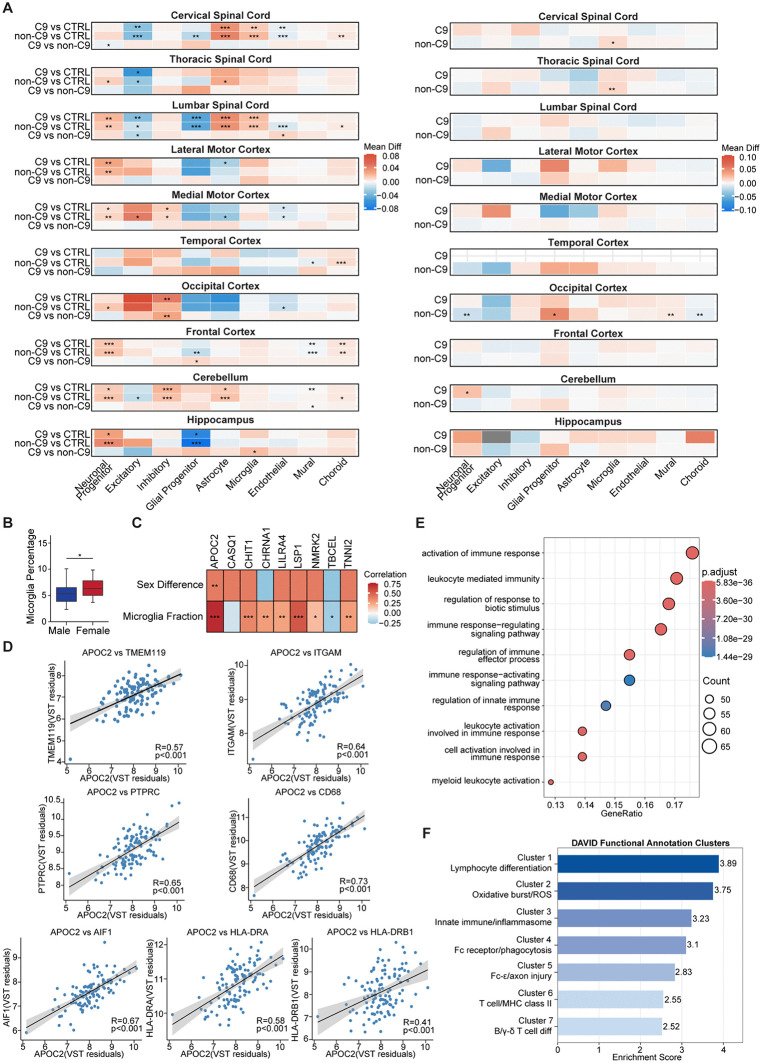
Shared cellular shifts in ALS subtypes with non-C9-specific microglial expansion and sex-biased gene dysregulation linked to poor outcomes. **(A)** The cell fraction differences of each cell type in ALS disease (left) and ALS clinical duration (right) are summarized across tissues. Comparisons in ALS disease are displayed for C9 patients vs. control, non-C9 patients vs. control, and C9 patients vs. non-C9 patients. While in ALS clinical duration, comparisons are shown for short- vs. long-duration group in C9 patients and non-C9 patients. The mean differences are presented by color gradient, with red presented an increase in patients (when C9 patients vs. control, non-C9 patients vs. control), C9 patients (when C9 patients vs. non-C9 patients) or short-duration group (when short- vs. long-duration group in C9 patients and non-C9 patients) and blue presented a decrease. Asterisks are labeled for statistical significance (* p-value < 0.05, ** p-value < 0.01, *** p-value < 0.001). **(B)** The box plot shows the difference of microglia proportion between female and male in cervical spinal cord. **(C)** The correlation between 9 clinical duration-related differentially expressed genes (DEGs) in the cervical spinal cord and microglia proportion, as well as sex. In the Sex Difference, red indicated DEGs with higher expression in female, while blue resents DEGs with higher expressed in male. Asterisks are labeled for statistical significance (* p-value < 0.05, ** p-value < 0.01, *** p-value < 0.001). **(D)** Correlation between *APOC2* expression and microglia-related markers genes in ALS-non-C9 cervical spinal cord samples. Scatter plots show the Pearson correlation between *APOC2* and individual microglia markers, including *TMEM119*, *CD11b*, *CD68*, *Iba1*, *HLA-DRA*, *HLA-DRB1* and *CD45*. **(E)** Dot plot of the top 10 significantly enriched GO terms based on adjusted p-value. Dot size indicates the number of genes in each term, and color reflects the statistical significance (adjusted p-value). **(F)** DAVID functional annotation clustering of *APOC2* direct co-expression neighbors. Horizontal bar chart displaying the enrichment scores of seven functional annotation clusters derived from DAVID analysis.

For cellular composition analysis in ALS disease (Fig 5A, left panel), we compared ALS-C9 patients and ALS-non-C9 patients separately against controls. Among neuronal cells, neuronal progenitor cells were increased in both ALS-C9 and ALS-non-C9 patients compared to controls across multiple regions, including the lumbar spinal cord, lateral motor cortex, medial motor cortex, frontal cortex, cerebellum, and hippocampus. However, the increase was observed in the thoracic spinal cord and occipital cortex only in ALS-non-C9 patients. Excitatory neurons were decreased in all spinal cord regions in both patient groups but an increase in the medial motor cortex and a decrease in cerebellum exclusively in ALS-non-C9 patients. Inhibitory neurons were elevated in both groups in the medial motor cortex and cerebellum, with an additional increase in the occipital cortex observed only in ALS-C9 patients.

Among glial cells, glial progenitor cell levels were reduced in the lumbar spinal cord and hippocampus in both patient groups, while significant reductions in the cervical spinal cord and frontal cortex were only detected in ALS-non-C9 patients. Astrocyte and microglia were increased in both patient groups in the cervical and lumbar spinal cord. Astrocyte levels were also elevated in the cerebellum in both ALS-C9 and ALS-non-C9 patients but showed a decrease in the lateral motor cortex of ALS-C9 patients and in the medial motor cortex of ALS-non-C9 patients.

For vascular cells, endothelial cells were decreased in the cervical spinal cord and medial motor cortex in both patient groups, with an additional decrease observed in the lumbar spinal cord and occipital cortex only in ALS-non-C9 patients. Mural cells were decreased, while choroid plexus cells were increased in the frontal cortex in both ALS-C9 and ALS-non-C9 patients. Additionally, mural cells were reduced in the temporal cortex in ALS-non-C9 patients and in the cerebellum in ALS-C9 patients. Choroid plexus cells also exhibited an increase in the cervical spinal cord, lumbar spinal cord, temporal cortex, and cerebellum exclusively in non-C9 patients.

We further compared cell type composition between ALS-C9 patients vs. ALS-non-C9 patients (Fig 5A, left panel). Neuronal progenitor cells in the cervical spinal cord and excitatory neurons in the lumbar spinal cord were less abundant in ALS-C9 patients than in ALS-non-C9 patients. In contrast, inhibitory neurons in the occipital cortex, glial progenitor in the frontal cortex, microglia in the hippocampus, endothelial cells in the lumbar spinal cord, mural cells in the cerebellum, were more abundant in ALS-C9 patients than in ALS-non-C9 patients.

For cellular composition analysis in ALS clinical duration (Fig 5A, right panel), we conducted two comparisons, short-duration versus long-duration within ALS-C9 patients and within ALS-non-C9 patients. Limited statistically significant differences in cell type composition were identified in relation to clinical duration. In the short-duration group, ALS-non-C9 patients exhibited a higher microglia fraction in the cervical spinal cord and thoracic spinal cord. In the occipital cortex, glial progenitor and mural cell fractions were increased, while neuronal progenitor and choroid cell fractions were decreased in ALS-non-C9 patients. In contrast, the only significant change in ALS-C9 patients was an increase in neuronal progenitor cells in the cerebellum in the short-duration group.

Given that female patients demonstrated worse survival outcomes than male patients within ALS-non-C9 patients (Fig 1E), we also examined the disease duration-related cell composition changes by sex (S9 Table). Our results showed that microglia fractions in the cervical spinal cord were associated with sex differences in ALS-non-C9 patients. Female ALS-non-C9 patients exhibited significantly higher microglia levels than their male counterparts (Fig 5B). This finding aligns with our observation that increased microglia levels correlate with short clinical duration in ALS-non-C9 patients (Fig 5A, right panel), suggesting that female ALS-non-C9 patients, who exhibited higher microglia levels, may experience shorter clinical duration than males.

We further investigated the associations of sex difference, microglia fraction, and the 9 DEGs identified in the cervical spinal cord of ALS-non-C9 patients associated with short disease duration. Among these, *APOC2*, *CHIT1*, *CHRNA1*, *LILRA4*, *LSP1*, *NMRK2*, *TBCEL*, and *TNNI2* exhibited positive correlation with microglia fractions. Notably, *APOC2*, which encodes a lipid binding protein, emerged as the most positively correlated gene with microglia fractions among the 9 DEGs. It was also the only DEG showing a significant sex difference, being upregulated in female ALS-non-C9 patients compared to male ALS-non-C9 patients (Fig 5C). We examined the correlation between *APOC2* expression levels and microglia activation marker levels (S10 Table). *APOC2* expression showed a positive correlation with microglia-specific markers *TMEM119* (R = 0.57, *p* = 3.18x10^-11^, *p.adj* = 4.24x10^-11^) and *CD11b* (R = 0.64, *p* = 1.94x10^-14^, *p.adj* = 3.88x10^-14^). In addition, activated microglia markers *CD45* (R = 0.65, *p* = 7.01x10^-15^, *p.adj* = 1.87x10^-14^), *CD68* (R = 0.73, *p* = 2.03x10^-20^, *p.adj* = 1.62x10^-19^), *HLA-DRA* (R = 0.58, *p* = 8.83x10^-12^, *p.adj* = 1.41x10^-11^), *HLA-DRB1* (R = 0.41, *p* = 5.83x10^-6^, *p.adj* = 6.67x10^-6^) and *Iba1* (R = 0.67, *p* = 5.25x10^-16^, *p.adj* = 2.10x10^-15^) were also positively correlated with *APOC2* expression (Fig 5D).

To gain insight into the biological function associated with the *APOC2* co-expression network, we constructed a weighted gene co-expression network analysis (WGCNA) using RNA-seq data from ALS-non-C9 cervical spinal cord samples. As *APOC2* was not assigned to any co-expression module in the WGCNA analysis, its expression in the cervical spinal cord appears to operate relatively independently of the major coordinated transcriptional programs captured by the network. Pathway enrichment analysis was therefore conducted using direct co-expression neighbors of *APOC2*, defined by Pearson correlation (r > 0.5, FDR < 0.05), yielding 412 co-expressed genes (S11 Table) that were subsequently subjected to Gene Ontology (GO) over-representation analysis (ORA) using the Biological Process category (S12 Table). The top 10 most significant enriched pathways were predominantly immune-related, collectively reflecting broad activation of innate and adaptive immune signaling. These included pathways governing leukocyte-mediated immunity, myeloid leukocyte activation, and cell activation involved in immune response, alongside regulatory programs such as regulation of innate immune response, regulation of immune effector processes, and regulation of response to biotic stimulus, as well as upstream signaling programs including immune response-regulating and immune response-activating signaling pathways (Fig 5E).

Functional annotation clustering of the 412 APOC2 co-expressed genes using DAVID revealed seven enriched clusters, all reflecting immune and inflammatory biological functions. The highest-scoring cluster was lymphocyte differentiation (enrichment score 3.89), followed by oxidative burst and reactive oxygen species signaling (3.75), and innate immune response and inflammasome activation (3.23). Additional clusters included Fc receptor-mediated phagocytosis (3.10), Fc receptor and axon injury response (2.83), T cell activation and MHC class II antigen presentation (2.55), and B cell and T cell differentiation (2.52). The consistent enrichment of immune-related clusters across all seven annotation groups indicates that *APOC2* co-expression in the cervical spinal cord is strongly embedded within a neuroinflammatory transcriptional context, with particular involvement of innate immune activation, myeloid cell function, and leukocyte-mediated cytotoxicity (Fig 5F), collectively suggesting that *APOC2*-associated genes participate in a tightly coordinated immune-regulatory network relevant to ALS-non-C9 disease duration in this region.

## Discussion

Our present study uncovers how genetic background and sex intersect to shape the molecular architecture and clinical duration-associated transcriptional dynamics of ALS. By leveraging multi-regional transcriptomic and cellular data, we identified both shared biological processes across ALS patients and distinct molecular features that differentiate ALS-C9 from ALS-non-C9 subtypes. Our analysis revealed disease-relevant pathways, stable versus transient molecular signatures, and cell-type composition changes associated with disease state and clinical duration. Herein, we discuss how these findings illuminate molecular mechanisms and support biologically meaningful stratification to advance our understanding of ALS pathophysiology.

A key hallmark of ALS is misfolding of disease-associated proteins and pathological aggregation of proteins such as TDP-43, SOD1, and p62, leading to an imbalance in cellular protein homeostasis [24–26]. In our study, pathways related to protein secretion and the unfolded protein response were consistently enriched across multiple CNS tissues in both ALS-C9 and ALS-non-C9 groups. This pattern underscores a shared vulnerability in protein handling mechanisms, suggesting that disrupted proteostasis is a convergent pathogenic axis across genetically distinct forms of ALS. The consistent activation of the unfolded protein response likely reflects a cellular attempt to alleviate proteotoxic stress associated with misfolded or aggregated proteins. Importantly, we also observed enrichment of the mTORC1 signaling pathway in both subtypes, aligning with its central role in regulating protein homeostasis. mTORC1 orchestrate critical processes such as autophagy, energy metabolism, and protein synthesis, while also influencing neuron survival [27]. When coupled with enrichment in protein secretion pathways, the involvement of mTORC1 further highlights the importance of maintaining a functional protein quality control system in motor neuron health.

Our analysis also highlights the potential involvement of androgen signaling in ALS, aligning with prior observations that androgen receptors (AR) are highly expressed in motor neurons and skeletal muscle. Experimental models of *SOD1* mutation have demonstrated that impaired AR signaling accelerates disease onset and exacerbates neuromuscular pathology [28,29]. Beyond proteostasis and hormonal regulation, dysregulation of cell cycle and DNA repair pathways also emerged as notable features in ALS-associated gene expression profiles. Aberrant activity of E2F target genes and G2/M checkpoint components may reflect affected neurons are attempting to re-entry the cell cycle inappropriately or are struggling to maintain genomic stability. Supporting this, mutations in DNA/RNA-binding protein such as FUS have been linked to impaired DNA repair capacity and are linked to early-onset form of ALS [30–32]. Additionally, altered KRAS signaling was observed, though its functional role in neurodegeneration remains poorly understood. Given its regulatory influence on multiple cellular processes, including growth and survival, KRAS-associated pathways may intersect with broader ALS pathophysiological mechanisms and deserve further investigation.

Despite shared features, our analysis also reveals divergent molecular pathway activations between ALS patients with C9orf72 (ALS-C9) and those without (ALS-non-C9), underscoring potential subtype-specific mechanisms of disease onset and disease duration. The ALS-C9 group demonstrates a distinct pattern of pathways enrichment suggestive of lipid homeostasis and immune modulatory. The pathological accumulation of cholesterol within macrophages and related myeloid cells serves as a potent driver of systemic inflammation. This lipid buildup actively primes the innate immune system by augmenting Toll-like receptor (TLR) signaling and triggering inflammasome activation, which subsequently accelerates the production of pro-inflammatory monocytes and neutrophils [33]. Within the central nervous system (CNS), the inability to effectively clear this surplus cholesterol is particularly detrimental, as lipid-induced toxicity contributes to neuronal demise and has been clinically linked to poorer patient prognosis [34]. Recent evidence underscores the existence of a regulatory feedback loop where TLR inhibition can actually restore lipid balance. The inhibition of Toll-like receptor (TLR) pathways has been shown to mitigate the development of lipid-congested foam cells by enhancing the expression of critical cholesterol efflux transporters, including ABCA1, ABCG1, and SR-B1. This regulatory mechanism likely operates through a coordinated molecular axis involving p65/ NFκB, LXR-α, and ABCA1 to maintain cellular lipid balance [35]. Our identified ALS-C9-specific transcriptional signature aligns with these mechanisms, as the C9 cohort demonstrated significant enrichment in cholesterol homeostasis and TLR pathway. This suggests that the convergence of lipid metabolic dysfunction and heightened immune reactivity represents a central molecular axis in C9orf72-mediated pathogenesis, distinguishing it from the metabolic attrition observed in non-C9 cases. Additionally, evidence of blood brain barrier dysfunction in C9 models [36] align with the observed upregulation of angiogenesis pathways, suggesting vascular instability. This vascular permeability likely facilitates peripheral immune cell infiltration [37], as reflected in the concurrent enrichment of leukocyte activation and adhesion pathway in ALS-C9 patients. Together, these findings point to a coordinated disruption of neurovascular and immune regulation uniquely associated with C9orf72 pathology, underscoring a distinct inflammatory and glial landscape in this genetic subtype of ALS.

Throughout our analysis of disease duration, the reference baseline was defined as long-duration patients and the comparison group was short-duration patients across both genotypes. Intriguingly, we observed divergent pathway trajectories between the two cohorts associated with disease duration. In ALS-C9 cases, shorter duration patients exhibited relatively higher enrichment of metabolic, proliferative, and immune-related pathways, such as MYC targets, mTORC1 signaling, and oxidative phosphorylation compared with long-duration patients. In contrast, ALS-non-C9 patients displayed the opposite pattern, with these pathways showing relatively lower enrichment in short-duration cases, suggesting potential differences in underlying pathogenic kinetics or cellular adaptive responses between these genetic subtypes.

The enrichment of protein synthesis-related pathways, particularly cytoplasmic translation and ribosomal assembly, among ALS-non-C9-specific DEGs aligns with a growing body of evidence implicating translational dysregulation as a central mechanism in ALS pathogenesis. Defective local translation has been proposed to underlie the neurodegenerative process in ALS, as the transport of mRNAs encoding ribosomal subunits to axons is critical for replenishing the local ribosomal pool, and disruption of this process leads to a shortage of proteins required for axonal maintenance. In ALS, the nuclear-to-cytoplasmic mislocalization and aggregation of RNA-binding proteins, including TDP-43 and FUS [38,39], have been shown to broadly disrupt cytoplasmic translation, with TDP-43 proteinopathy specifically altering the ribosome association of multiple mRNA targets [40]. The transcriptional downregulation of ribosomal assembly and cytoplasmic translation programs observed in this ALS-non-C9 may reflect the downstream consequences of dysfunction on the translational machinery.

The overlap of the ALS-non-C9-specific signature with Alzheimer’s, Parkinson’s, and Huntington’s diseases further supports the notion that translational dysregulation represents a shared molecular vulnerability across neurodegenerative conditions. Defective mRNA localization, inhibited ribosome biogenesis, and translational inhibition have each been implicated across ALS, FTD, Alzheimer’s disease, and Huntington’s disease as convergent pathomechanisms [41], suggesting that ribosomal integrity and cytoplasmic translation capacity constitute a broader axis of neuronal vulnerability. The observation that ALS-non-C9 shares this generalized neurodegenerative transcriptional landscape, while ALS-C9 exhibits a more genotype-specific immune and lipid-driven signature, highlights the distinct molecular contexts in which motor neuron degeneration unfolds across ALS genetic subgroups.

ALS exhibits notable sex-related differences in incidence, clinical presentation, and disease duration [42]. While some studies report faster respiratory decline and reduced survival in males, others highlight a more rapid functional deterioration in females, as measured by the ALS Functional Rating Scale-Revised (ALSFRS-R) [43]. Moreover, women also show a higher prevalence of bulbar-onset ALS [44,45], a clinical phenotype linked to accelerated cognitive decline, diminished quality of life, and shorten survival [46,47]. Consistent with these observations, our finding show that the female ALS patients experienced shorter survival than males, both in the overall cohort and within the ALS-non-C9 subgroup. These sex-related disparities may be partly driven by sex-dependent immunology and glial responses, as suggested by recent studies [48,49].

Motor neurons and microglia have been implicated in ALS onset and progression, as demonstrated *in vivo* using an *SOD1* mutant-induced ALS mouse model [12]. Furthermore, neurovascular impairment has been identified in various neurodegenerative diseases, including ALS [50]. Emerging mechanistic insights implicate microglia activation as a potential driver of these sex-specific differences in disease progression. Microglia-mediated neuroinflammation has been shown to contribute directly to motor neuron degeneration through NFkB signaling in ALS model [51], and elevated markers of activated microglia (CD68 and Iba1) have been associated with faster disease progression [52,53]. In our study, microglia enrichment was notably elevated in the cervical spinal cord of fast-progressing ALS-non-C9 patients compared to those with slow processing. In contrast, no such differences observed in ALS-C9 patients. This may be attributed to prior findings that C9orf72 repeat expansions suppress microglial activity, potentially blunting differential responses across progression subgroups [54].

Moreover, microglial phenotypes are known to modulate the neuroinflammatory landscape and are closely associated with disease pathology, particularly in neurodegenerative disorders such as Alzheimer’s disease and Parkinson’s disease [55], underscoring their critical role in disease trajectory. Pro-inflammatory M1 microglia induce neuroinflammation, while M2 microglia promote neuroprotection. This polarization is influenced by both intrinsic and extrinsic factors, including sex hormones [56,57]. Extracellular α-synuclein, a pathogenic protein linked to the Parkinson’s disease, has been shown to promote microglia polarization toward a pro-inflammatory M1 state, with stronger effects observed in postmenopausal women duo to reduce estrogen and its loss of neuroprotective capacity [58]. Given that average age of our ALS cases was around 60 years, likely reflecting a postmenopausal population, our findings raise the possibility that diminished estrogen may heighten susceptibility to microglia-driven neuroinflammation. This may contribute to the poor prognosis observed in females, particularly in the ALS-non-C9 subgroup where microglial activation appears more pronounced. Collectively, these data suggest that sex-linked differences in microglial dynamics may underlie, at least in part, the divergent clinical outcomes observed in ALS, emphasizing the need for sex-specific therapeutic strategies targeting neuroinflammation.

Several limitations of the present study warrant consideration. First, cell type deconvolution was performed using a reference dataset derived from cortical tissue, which may not fully capture the cellular composition of anatomically distinct regions such as the spinal cord. Spinal cord-specific cell populations, including ependymal cells and region-specific interneuron subtypes, are not adequately represented in cortical reference panels, potentially leading to incomplete or imprecise deconvolution estimates in these regions. This limitation should be considered when interpreting cell type proportion estimates in spinal cord tissues, as the absence of relevant reference signatures may introduce systematic bias in the decomposition of bulk RNA-seq signals. Second, the sample size available for certain tissues in the ALS-C9 group was limited, most notably the temporal cortex (n = 1), which precluded reliable statistical inference in these regions. While findings from well-represented tissues are supported by robust sample sizes, conclusions drawn from low-sample regions in the ALS-C9 group should be regarded as preliminary and interpreted with appropriate caution. The inherent rarity of C9orf72 expansion carriers among ALS cases and the constraints of existing postmortem biobanks present ongoing challenges for expanding these cohorts, and future studies with larger region-matched ALS-C9 sample sizes will be needed to validate and extend the observations reported here.

## Conclusion

In conclusion, ALS patients share core pathogenic pathways, particularly those related to protein homeostasis, cellular responses, and metabolic regulation, distinct molecular signatures and divergent progression dynamics are also observed between these genetic subtypes. ALS-C9 is characterized by a more pronounced activation of immune and stress-related pathways in the short-duration groups than the long-duration group. In contrast, ALS-non-C9 patients exhibit a distinct regulatory pattern, where the lower enrichment of metabolic and biosynthetic pathways in short-duration cases compared to long-duration individuals likely reflects the diverse genetic and environmental etiologies underlying ALS-non-C9. Microglia activation emerged as a potential modifier of disease progression, especially in ALS-non-C9 patients, with sex-specific influences suggesting that the loss of estrogen after menopause may amplify microglia-driven neuroinflammation and worsen outcomes in female patients. The fact that the *C9orf72* repeat expansion is the most common cause of both ALS and FTD underscores the shared molecular continuum that bridges motor and cognitive neurodegeneration. Our findings highlight that genetic context, glial-neuronal interactions, and sex biology collectively shape disease heterogeneity, and these principles are likely to extend across neurodegenerative disorders.

## Materials and methods

### Study samples

In this study, we utilized 1,146 RNA-seq profiles generated from 316 de-identified post-mortem samples provided by New York Genome Center (NYGC) ALS Consortium. The data that support the findings of this study are publicly available from the Gene Expression Omnibus (GEO) with the identifiers GSE137810, GSE124439, GSE116622 and GSE153960, and can also be accessed via DOI 10.1038/s41586-022-04424-7. Samples originally adapted from cortical regions, cerebellum and spinal cord across central nervous system. Clinical and pathological information were also acquired from NYGC ALS Consortium, as well as *C9orf72* genotypes. ALS-C9 and ALS-non-C9 group were defined based on the genotypes of *C9orf72*.

### Survival analysis

Survival analysis was performed to assess the correlation between clinical and genetic factors, and disease progression in ALS patients. Survival duration was defined as the time interval between symptom onset age and age at death. Kaplan-Meier Curves were applied to visualize survival distribution. Cox proportional hazards regression models were applied to estimate hazard ratios (HRs) and the influence of covariates. All statistical analyses were performed using the survival package in R (version 4.3.1). Statistical significance was defined as a Benjamini-Hochberg (BH) adjusted  p-value ≤ 0.05 to account for multiple testing.

### Differentially express genes analysis

Differential analysis of count data was performed in each tissue separately using the DESeq2 R package (version 1.40.2). To evaluate the impact of disease condition while accounting for *C9orf72* repeat expansion status, generalized linear model was constructed with distinct parameterizations for disease status and disease duration.

For the disease status analysis, the independent variable included three groups: control, *C9orf72*-positive ALS, and *C9orf72*-negative ALS, with each ALS subtype compared directly against the control group. For the disease duration analysis, differential expression was modeled within the *C9orf72*-positive ALS and *C9orf72*-negative ALS cohorts separately, evaluating the contrast between short and long disease durations.

A linear model between a molecular feature (as a dependent variable) and these respective independent variables was fitted by including clinical variables (sex and age at death), library preparation methods and sequencing quality metrics (RIN) as covariates. Categorical covariates with zero variance within a given tissue sub-cohort were dynamic omitted. Statistical significance was determined at a Type I error rate of α = 0.05.

### Sex-by-diagnosis interaction analysis

Given the unequal sex distribution across diagnostic groups, we examined whether sex could differentially modify the transcriptomic response to ALS diagnosis in a region- and group-specific manner. For each tissue, an interaction model was constructed within the DESeq2 framework, extending the standard covariate structure of diagnosis, sex, age, RIN, and library preparation method to include an explicit sex-by-diagnosis interaction term. Genes whose interaction term reached statistical significance after multiple testing correction (adjusted p-value < 0.05) were classified as exhibiting sex-dependent expression patterns, and the proportion of such genes among all tested genes was calculated for each tissue-group combination to gauge the overall extent of sex-driven confounding.

### Sensitivity analysis

To quantify the degree to which unobserved sex-related confounding could undermine the ALS-associated transcriptomic findings, we conducted a formal sensitivity analysis using the sensemakr R package. For each tissue and diagnostic group, the top 10 differentially expressed genes ranked by adjusted p-value were taken forward for evaluation. At the gene level, the robustness value with respect to a quarter of the residual variance (RV q1) was derived for each candidate gene, reflecting the minimum confounding strength required to render the observed effect statistically negligible. A gene was designated robust when its RV q1 surpassed a pre-specified threshold, and the percentage of robust genes among the top 10 was recorded per tissue. All sensitivity evaluations were performed independently for ALS-C9 and ALS-non-C9 groups across all tissues with sufficient sample representation.

### Gene set and pathway enrichment analysis

We performed gene set enrichment analysis (GSEA) and over-representation analysis (ORA) to identify significant enrichment pathways and gene sets within expression data. To enhance computational efficiency, we utilized the Fast Gene Set Enrichment Analysis (FGSEA) algorithm, as implemented in the fgsea R package (version 1.26.0). To ensure that both the magnitude and direction of differential expression were captured in the GSEA input, all genes were ranked using the signed Wald statistic derived from DESeq2’s results() function, defined as the log2 fold change divided by its standard error. This metric ensures that genes with large positive fold changes and high statistical confidence receive the highest ranks, while those with large negative fold changes and high confidence receive the lowest ranks, thereby enabling a precise interpretation of the normalized enrichment score (NES). For the analysis of specific genes, ORA was performed using the clusterProfiler R package (version 4.8.3), with the gene list as input. Pathways and gene sets annotations were sourced from multiple curated databases, including the Hallmark gene sets, Kyoto Encyclopedia of Genes and Genomes (KEGG) gene sets, and Gene Ontology (GO) Biological Process (BP), Cellular Component (CC) and Molecular Function (MF) gene sets. Pathway were considered significant enriched at a threshold of adjusted p-value < 0.05, with the direction of enrichment assessed using the normalized enrichment score (NES), where NES > 0 indicated positively enrichment and NES < 0 indicated negatively enrichment.

### Dispersion estimation and model diagnostics

To examine tissues with atypically high DEG counts, a two-part diagnostic evaluation was conducted following the primary differential expression analysis. First, the adequacy of the DESeq2 negative binomial model fit was assessed by inspecting gene-wise dispersion estimates plotted against mean normalized expression levels, with the fitted shrinkage trend and final posterior estimates overlaid to evaluate whether dispersion regularization behaved as expected across the expression range. Systematic deviations between gene-level and shrunken estimates, particularly at low mean counts, were used as indicators of potential model instability or outlier-driven inflation. Second, to characterize sample-level variation and assess between-group separation, expression profiles were stabilized using variance-stabilizing transformation with blind = FALSE to preserve experimental group structure, and the resulting matrix was subjected to principal component analysis. The distribution of samples along the primary principal components was examined in relation to clinical duration group assignments to determine whether observed DEG inflation could be attributed to transcriptomic outliers or insufficient cohort-level separation rather than a genuine biological signal.

### Cell deconvolution

To characterizing the cell composition of bulk RNA-seq data, we employed CIBERSORTx [59] using a reference single-cell RNA sequencing (scRNA-seq) transcriptome derived from human cerebral cortex [23]. Gene expression count data were normalized to transcripts per million (TPM) using cout2tpm function implemented in the IOBR R package. Cell types were categorized into ten major groups: neuronal progenitor, excitatory neuron, inhibitory neuron, glial progenitor, astrocyte, microglia, endothelial cells, mural cells, choroid plexus cells, and unclassified category. Difference in cell fraction distributions between groups were assessed using the Kruskal-Wallis test followed by post-hoc Dunn’s test for comparisons among three groups, and the Wilcoxon rank-sum test for pairwise comparisons. Statistical significance was set at a threshold of p-value ≤ 0.05.

### Co-expression network analysis

To construct the gene co-expression network, we utilized the weighted gene co-expression network analysis (WGCNA) framework by using the WGCNA R package (version 1.72.5). RNA-seq raw count data were first transformed using the variance stabilizing transformation (VST) method implemented in the DESeq2 R package (version 1.40.2). Prior to network construction, the effects of potential confounding covariates, including age, sex, RNA integrity number (RIN), and library preparation method, were regressed out from the VST-normalized expression matrix using a linear model, and the resulting residuals were used as input for subsequent network construction. Soft-thresholding power was chosen based on the scale-free topology criterion to emphasize strong correlations and reduce weak ones. The resulting adjacence matrix was transformed into topological overlap matrix (TOM), which accounts for both direct and indirect interactions. Genes were hierarchically clustered based on TOM dissimilarity, and modules were identified using the dynamic tree cut algorithm with a minimum module size of 30 genes. Direct co-expression neighbors were also identified for gene of interest based on a Pearson correlation threshold of r > 0.5 with FDR < 0.05, and the resulting neighbor gene sets were subjected to Gene Ontology pathway enrichment analyses using the clusterProfiler R package (version 4.8.3) to infer potential biological process and pathway involvement, complemented by functional annotation lustering via DAVID [60,61].

## Supporting information

S1 Table Basal characteristics of subjects from NYGC. (XLSX)

S2 Table Differential express genes (DEGs) counts for ALS disease. (XLSX)

S3 Table Analysis of sex-by-diagnosis interaction effects across multiple CNS tissues. (XLSX)

S4 Table Sensitivity analysis of the top 10 differentially expressed genes (DEGs) across CNS tissues in ALS-C9 and ALS-non-C9 cohorts. (XLSX)

S5 Table ALS-C9 Specific differential genes expression (Log 2 Fold change) across CNS tissues. (XLSX)

S6 Table ALS-non-C9 Specific differential genes expression (Log 2 Fold change) across CNS tissues. (XLSX)

S7 Table Differential express genes (DEGs) counts for ALS clinical duration. (XLSX)

S8 Table Enrichment trend of Gene set enrichment analysis for ALS disease and ALS duration. (XLSX)

S9 Table Association between cell fraction and sex. (XLSX)

S10 Table Correlation between *APOC2* and microglia markers. (XLSX)

S11 Table *APOC2* direct co-expression neighbors identified in the cervical spinal cord of ALS-non-C9 patients. (XLSX)

S12 Table GO enrichment analysis of *APOC2* co-expressed genes in the ALS-non-C9 cervical spinal cord. (XLSX)

S1 Fig Tissue-wide distribution of differentially expressed genes in ALS-C9 and ALS-non-C9. Bar plot showing the number of differentially expressed genes (DEGs) identified in each tissue for ALS-C9 (red) and ALS-non-C9 (blue) groups. The horizontal axis represents the total number of DEGs detected.
(TIF)

S2 Fig Bootstrap permutation analysis of DEG counts across tissues. To evaluate whether the observed differential expression signals between ALS and control groups could be attributed to sample size discrepancy between ALS-C9 and ALS-non-C9 cohorts, a bootstrap permutation procedure was performed with 100 iterations, in which equivalent numbers of samples were drawn from each group to match the smaller ALS-C9 cohort. Each panel displays the null distribution of DEG counts generated across 100 bootstrap iterations (blue histogram and density curve) for each tissue, with the observed DEG count indicated by the red vertical line. Bootstrap analysis was not performed for the temporal cortex in the ALS-C9 group due to insufficient sample size (n = 1). Tissues are arranged by anatomical region, encompassing (A) cervical spinal cord, (B) lumbar spinal cord, (C) thoracic spinal cord, (D) lateral motor cortex, (E) medial motor cortex, (F) frontal cortex, (G) cerebellum, (H) hippocampus, (I) occipital cortex. Red lines falling within or to the right of the bootstrapped distribution indicate that the observed DEG count is consistent with or exceeds what would be expected under sample size equalization, whereas red lines falling at the left tail suggest attenuated differential expression signal in that region.
(TIF)

S3 Fig Cross-tissue overlap of differentially expressed genes in ALS-C9 and ALS-non-C9. UpSet plot illustrating the intersection of differentially expressed genes (DEGs) associated with (A) ALS-C9 case-control and (B) ALS-non-C9 case-control across cervical spinal cord, thoracic spinal cord, lumbar spinal cord, lateral motor cortex, medial motor cortex, temporal cortex, occipital cortex, frontal cortex, and cerebellum. Upregulated (top) and downregulated (bottom) DEGs are displayed separately. Vertical bars present the size of overlapping gene sets across tissues. The connected dots below the vertical bar represent the combinations across tissues.
(TIF)

S4 Fig DESeq2 diagnostic plots for Cerebellum ALS-C9 clinical duration analysis. Dispersion estimation plot for the Cerebellum ALS-C9 cohort. Gene-wise dispersion estimates (black dots) are shown as a function of mean normalized counts, with the fitted trend line (red) and final shrunken dispersion estimates (blue) overlaid. The convergence of final estimates toward the fitted curve across the range of mean expression values indicates appropriate model fitting without evidence of systematic overdispersion or model misspecification.
(TIF)

S5 Fig Pathway transition landscape in ALS tissues. Sankey diagrams illustrate transitions in pathways enrichment specificity across the axes of ALS disease state specificity, pathway identity, and disease duration specificity across and (A) frontal cortex, (B) lumbar spinal cord, (C) cerebellum, (D) occipital cortex, (E) thoracic spinal cord, and (F) medial motor cortex, (G) hippocampus, (H) temporal cortex. Pathways are categorized into three specificity groups based on their enrichment profiles: C9-specific, non-C9-specific, or shared (significant in both groups). The direction of NES is visualized through color-coded flows: red for up regulation (positive NES), blue for downregulation (negative NES), light green for ALS-C9 downregulation but ALS-non-C9 upregulation, purple for ALS-C9 upregulation but ALS-non-C9 downregulation.
(TIF)
